# A Fast Sparse Decomposition Based on the Teager Energy Operator in Extraction of Weak Fault Signals

**DOI:** 10.3390/s22207973

**Published:** 2022-10-19

**Authors:** Baokang Yan, Zhiqian Li, Fengqi Zhou, Xu Lv, Fengxing Zhou

**Affiliations:** 1 Engineering Research Center for Metallurgical Automation and Measurement Technology of Ministry of Education, Wuhan University of Science and Technology, Wuhan 430081, China; 2 School of Artificial Intelligence, Wuchang University of Technology, Wuhan 430223, China

**Keywords:** fault diagnosis, filtered Teager energy operation, sparse decomposition, signal reconstruction

## Abstract

In order to diagnose an incipient fault in rotating machinery under complicated conditions, a fast sparse decomposition based on the Teager energy operator (TEO) is proposed in this paper. In this proposed method, firstly, the TEO is employed to enhance the envelope of the impulses, which is more sensitive to frequency and can eliminate the low-frequency harmonic component and noise; secondly, a smoothing filtering algorithm was adopted to suppress the noise in the TEO envelope; thirdly, the fault signal was reconstructed by multiplication of the filtered TEO envelope and the original fault signal; finally, sparse decomposition was used based on a generalized S-transform (GST) to obtain the sparse representation of the signal. The proposed preprocessing method using the filtered TEO can overcome the interference of high-frequency noise while maintaining the structure of fault impulses, which helps the processed signal perform better on sparse decomposition; sparse decomposition based on GST was used to represent the fault signal more quickly and more accurately. Simulation and application prove that the proposed method has good accuracy and efficiency, especially in conditions of very low SNR, such as impulses with anSNR of −8.75 dB that are submerged by noise of the same amplitude.

## 1. Introduction

The rotating components of machinery usually tend to manifest failures such as scuffing, pitting, and cracking. Detection of these failures in their incipient period can curb fault development, adjust production plans, and avoid huge economic losses and casualties [1,2]. The impulse component of the vibration signals from machinery is more insidious and harder to analyze, the main reasons being that these impulses usually hide in strong noise and appear non-periodic and sparse. Especially under the conditions of varying rotation speed, the fault type is impossible to identify through the traditional use of characteristic frequency [3].

In trying to solve the problem of impulse extraction, traditional fast Fourier transform (FFT) can only present the energy distribution of modulation frequency [4]. Methods such as wavelet transform (WT), empirical mode decomposition (EMD), local mean decomposition (LMD), and ensemble local mean decomposition (ELMD) are obviously effective in extracting signals with local features and widely used in mechanical fault diagnosis [5,6]. Meanwhile, many proven methods have been introduced to improve algorithm adaptability in signals with very low signal-to-noise ratio (SNR) [7]. Zhang proposed an optimized local mean ensemble decomposition method to determine an optimum set of ELMD parameters for vibration signal analysis, which can reduce mode mixing and improve fault detection ability [8]. Wang proposed a method based on ELMD and fast kurtograms to extract characteristic fault information from a strong noise-embedded signal [9]. Moreover, methods based on analytic models are also useful in incipient fault diagnosis, for example Wu established a dynamic model for a squirrel-cage induction motor in a dq coordinate system that can analyze the models and characteristics of an incipient broken-rotor-bar fault or turn-to-turn short fault [10]. To achieve more accurate representation of these impulses, Mallat first proposed a sparse representation method to extract impulses from an assembled redundant dictionary, which was a milestone of digital signal processing, and can represent impulses with higher precision [11]. The decomposition speed is the obvious deficiency, and to overcome this shortcoming, Yan proposed a fast sparse decomposition method based on time–frequency spectrum segmentation (SD-TFSS), which takes the complex matching pursuit to time–frequency spectrum processing [12]. Zhang proposed a whale optimization algorithm-optimized orthogonal matching pursuit (OMP) with a combined time–frequency atom dictionary, which can optimize the atomic parameters for best approximating the original signal [13].These methods are sensitive to noise, threshold, and decomposition level. In order to improve the SNR of the fault signal, Teager and Kaiser proposed an energy measurement method that includes both the amplitude and frequency of the signal [14,15,16]. This measure is called the Teager energy operator (TEO) [17]. However, in adopting the TEO, it is impossible to analyze a fault signal with varying speed, for example as in blind source separation and signal fault type identification.

In view of the above analysis, we proposed a novel fault diagnosis method using the TEO and sparse decomposition based on multi-resolution GST for rotating machinery in complicated conditions. In this method, we explored the structure of a signal processed by the TEO. Then, we proposed a preprocessing method with a filtered TEO to enhance the incipient fault signals, which can suppress noise and enhance impulses. Next, we used sparse decomposition based on multi-resolution GST to obtain the sparse representation of the enhanced signals. Finally, we rebuilt the initial fault signals and acquired the sparse representation of the original fault signals. Simulation and application verified the validity of the proposed method.

This paper is structured as follows: a fundamental introduction to the TEO and sparse decomposition based on multi-resolution GST time–frequency spectrum is presented in Section 2. The method proposed in this paper is presented in Section 3. The performance of the proposed method with simulated signals and engineering application is shown in Section 4 and Section 5. Finally, conclusions are presented in Section 6.

## 2. Background Theory

### 2.1. Teager Energy Operator

For any continuous signal x(t), the definition of the Teager energy operator is given by:

(1) $$\Psi [x(t)]={\left(\frac{dx(t)}{dt}\right)}^{2}-x(t)\left(\frac{d^{2}x(t)}{dt^{2}}\right)={\left[x^{\prime }(t)\right]}^{2}-x(t)x^{\prime \prime }(t)$$

where Ψ[x(t)] is the Teager energy operator of x(t), $x^{\prime }(t)$ is the first derivative of x(t) with respect to time t, and $x^{\prime \prime }(t)$ is the second derivative of x(t) with respect to time t.

If x(t) is a simple harmonic vibration signal, Ψ[x(t)] can track the energy of the signal. For example, if the system is set as an undamped linear positive vibration system constituted by a mass block and constant-stiffness spring, according to Newton’s second law:

(2) $$mx^{\prime \prime }(t)-kx(t)=0$$

where m is the mass of the mass block, k is the stiffness of the spring, x(t) is the displacement of the oscillator, and $x^{\prime \prime }(t)$ is the acceleration of the oscillator. So,

(3) x(t)=Acos(wt+φ)

where A is the amplitude of vibration, w is the natural angular frequency, and $w=\sqrt{k/m}$, φ is the initial phase.

The energy of the system contains the kinetic energy of the mass block and potential energy of the spring, it can be calculated by:

(4) $$E=\frac{1}{2}k{\left[x(t)\right]}^{2}+\frac{1}{2}m{\left[x^{\prime }(t)\right]}^{2}=\frac{1}{2}mA^{2}w^{2}$$

which means the instantaneous energy of the simple harmonic vibration system is proportional to the squared product of the amplitude and frequency [18].

According to Equations (1) and (3), the Teager energy operator is:

(5) $$\Psi [x(t)]={\left[x^{\prime }(t)\right]}^{2}-x(t)x^{\prime \prime }(t)=A^{2}w^{2}$$

The Teager energy operator can also reflect the energy of the signal x(t), and it is also valid for damping the vibration signal of the form

(6) $$x(t)=e^{-at}\cos(wt+\varphi_{0})$$

Furthermore, the first derivative of the Teager energy operator is given by

(7) $$\Psi [x^{\prime }(t)]={\left[x^{\prime \prime }(t)\right]}^{2}-x^{\prime }(t)x^{\prime \prime \prime }(t)=A^{2}w^{4}$$

which shows $\Psi [x^{\prime }(t)]$ is more sensitive to frequency than Ψ[x(t)], and it is more effective to improve the SNR of an impulse fault signal [19].

### 2.2. Sparse Decomposition Based on Multi-Resolution GST Time–Frequency Spectrum

**Figure 1:**
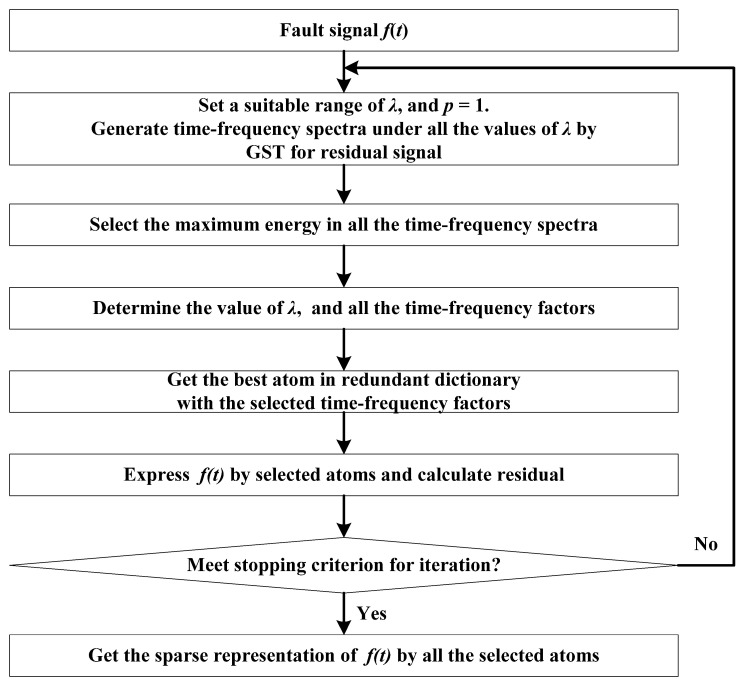
The flow of sparse decomposition based on a multi-resolution GST time–frequency spectrum.

In order to represent the signals with local features, the method of sparse decomposition adopts non-orthogonal and redundant atoms to match these signals, and it can achieve the representation more accurately and sparsely. However, to improve the accuracy of sparse decomposition, the scale of over-complete atom libraries is often very large, which seriously affects the efficiency of the search for the best atoms. Sparse decomposition based on a multi-resolution GST time–frequency spectrum can improve the efficiency of sparse decomposition by converting the atomic search process to an optimal time–frequency spectrum search. The flow of sparse decomposition based on a multi-resolution GST time–frequency spectrum is shown in Figure 1.

Realization of this method includes four steps: generation of all time–frequency spectra by GST, determination of optimal time–frequency factors, construction of optimal atoms by selected optimal time–frequency factors, and sparse representation using selected atoms.

In order to match the fault impulse signal better and facilitate the use of the fast algorithm, this method improves the model of impulse as:

(8) $$s(t)=Ae^{-\frac{\lambda^{2}f^{2}{\left(t-u\right)}^{2}}{2}}\cos(2\pi ft+\varphi )$$

where A is the amplitude,λ is the scale factor,u is the shift factor,φ is the phase factor, and f is frequency factor.

GST is defined as follows:

(9) $$\mathrm{GST}(\tau ,f)=\int_{-\infty }^{\infty }x(t)\frac{\lambda {\left|f\right|}^{p}}{\sqrt{2\pi }}e^{-\frac{\lambda^{2}f^{2p}{\left(\tau -t\right)}^{2}}{2}}e^{-i2\pi ft}dt$$

where *p* is the adjustment factor.

Set the model of the over-complete atom library as:

(10) $$g(t)=\pi^{-\frac{1}{4}}\lambda^{\frac{1}{2}}f^{\frac{1}{2}}e^{-\frac{\lambda^{2}f^{2}{\left(t-u\right)}^{2}}{2}}\cos(2\pi ft+\varphi )$$

Thus, ‖g(t)‖=1.

It is not difficult to prove that

(11) $$\left|<s(t)\;,g(t)>\right|=\sqrt{2}\pi^{\frac{1}{4}}\lambda^{-\frac{1}{2}}f^{-\frac{1}{2}}\mathrm{real}(\mathrm{GST}{\left(u,\lambda ,f\right)}_{p=1})$$

where $\mathrm{real}(\mathrm{GST}{\left(u,\lambda ,f\right)}_{p=1})$ is the real part of $\mathrm{GST}{\left(u,\lambda ,f\right)}_{p=1}$.

According to the analysis above, the inner product operation in atom searching can convert to searching for normalized time–frequency spectra with optimal λ. The maximum in the inner product corresponds to the maximum energy in the time–frequency spectra, and the time–frequency factors corresponding to the maximum energy are the optimal time–frequency factors of sparse decomposition.

Suppose *k* is the number of extracted optimal atoms, then the sparse representation of the signal f(t) is:

(12) $$f_{k}(t)=\sum_{i=1}^{k}<f(t),g_{i}>g_{i}$$

where f(t) is the analyzed signal, $f_{k}(t)$ is the sparse represented signal, $g_{i}$ is the atom, and $\left\|g_{i}\right\|=1$ [20].

## 3. Fault Diagnosis through Sparse Decomposition Based on the Teager Energy Operator

### 3.1. Signal Preprocessing through Filtered TEO

Equations (5)–(7) show the results of the TEO when the amplitude is the form of $e^{-at}$. Now if the amplitude shown in Equation (8) is identified as the form of $e^{-at^{2}}$, the TEO can also be computed as follows.

Set the damping vibration signal of the form

(13) $$x(t)=A_{1}\cos(wt+\varphi )$$

where $A_{1}=Ae^{-\frac{\lambda^{2}f^{2}{\left(t-u\right)}^{2}}{2}}$.

Thus, the expression of the TEO is

(14) $$\Psi [x(t)]=A_{1}^{2}w^{2}\left[1+\frac{2a}{w^{2}}\cos^{2}(wt+\varphi )+\frac{at}{w}\sin(2wt+2\varphi )\right]$$

As the impulse signal possesses the feature of rapid decay, extremely short duration, and high oscillation frequency, the TEO of x(t) and the first derivative of the TEO can be similarly expressed by

(15) $$\begin{array}{l} \Psi (t)=\Psi [x(t)]\approx A_{1}^{2}w^{2}\\ \Psi^{\prime }(t)=\Psi [x^{\prime }(t)]\approx A_{1}^{2}w^{4} \end{array}$$

Equation (15) shows both the TEO and the first derivative of the TEO are sensitive to frequency, so they are suitable for tracking impulse energy. Their disadvantage is that their shape would be affected by high frequency noise, so a smoothing filtering algorithm was adopted to maintain the shape. The operation is expressed by

(16) $$\begin{array}{l} \Psi_{s}(t)=\frac{1}{2k+1}\sum_{i=-k}^{k}\Psi (t+i)\\ {\Psi^{\prime }}_{s}(t)=\frac{1}{2k+1}\sum_{i=-k}^{k}\Psi^{\prime }(t+i) \end{array}$$

where $\Psi_{s}(t)$ and ${\Psi^{\prime }}_{s}(t)$ are the filtered TEO and filtered first derivative of the TEO, respectively.

**Figure 2:**
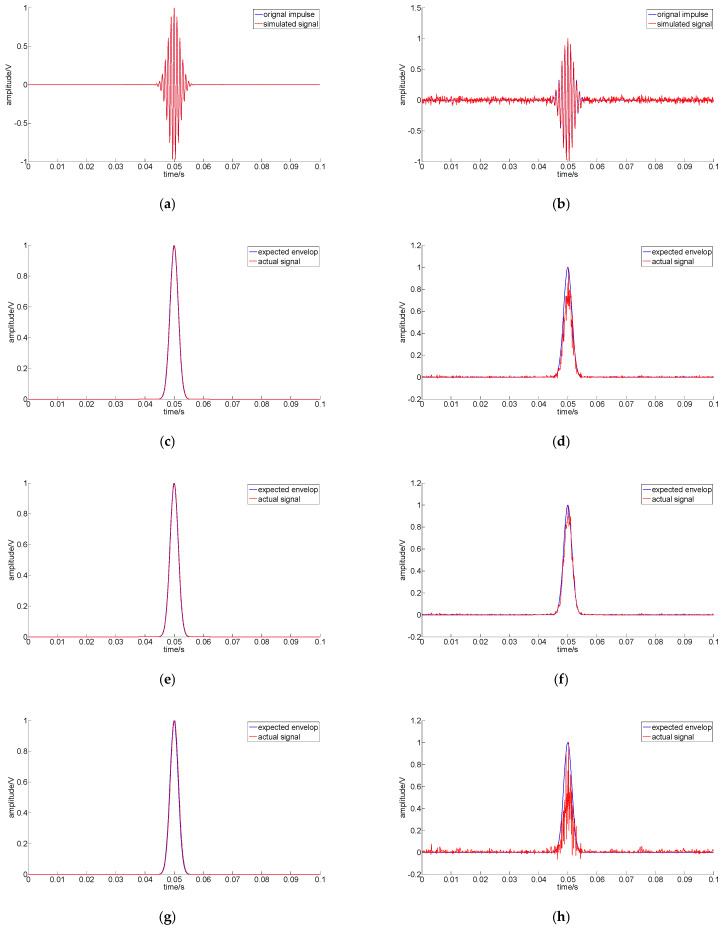

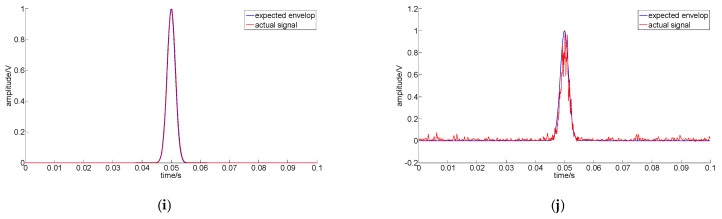
The TEO results of a noise-free impulse signal and a noisy impulse signal: (**a**) noise-free impulse signal; (**b**) impulse signal with noise; (**c**) normalized TEO result of (**a**); (**d**) normalized TEO result of (**b**); (**e**) normalized filtered TEO result of (**a**); (**f**) normalized filtered TEO result of (**b**); (**g**) normalized first derivative of the TEO result of (**a**); (**h**) normalized first derivative of the TEO result of (**b**); (**i**) normalized filtered first derivative of the TEO result of (**a**); (**j**) normalized filtered first derivative of the TEO result of (**b**).

Figure 2 shows the TEO results of a noise-free impulse signal and a noisy impulse signal.

Figure 2a,c,e,g,i shows that the four TEO operations can achieve the same impulse envelope structure for a noise-free impulse signal. Figure 2b,d,f,h,j shows that the four TEO operations can improve the amplitude of the impulse and suppress noise effectively, but Figure 2d,h show that the envelopes with the TEO and first derivative of the TEO are sensitive to high-frequency noise. Figure 2f,j shows that the filtered operation can maintain the structure of the envelope effectively, and the filtered TEO performs better than the filtered first derivative of the TEO. Therefore, in this paper, the filtered TEO was adopted to enhance impulse envelopes.

The main purpose of signal preprocessing in this paper was to enhance the impulses and suppress noise without damaging the signal structure. Therefore, for the proposed signal preprocessing method, firstly, obtain the impulse envelope through the TEO; secondly, obtain the enhanced envelope through the smoothing filtering algorithm; and thirdly, acquire the enhanced impulses through the product of the enhanced envelope and the original signal.

The preprocessed signal with the filtered TEO is expressed by

(17) $$s_{\Psi }(t)=\Psi_{s}(t)\cdot s(t)\approx A^{3}{\left(2\pi f\right)}^{2}e^{-\frac{3\lambda^{2}f^{2}{\left(t-u\right)}^{2}}{2}}\cos(2\pi ft+\varphi )$$

Thus, once the optimal time–frequency factors $\left(\lambda_{opt},f_{opt},u_{opt},\varphi_{opt}\right)$ can be extracted by sparse decomposition, the fault impulses could be restructured with factors of $\left(\lambda_{opt}/\sqrt{3},f_{opt},u_{opt},\varphi_{opt}\right)$.

### 3.2. Sparse Decomposition Based on the TEO

After preprocessing through filtered TEO method, the impulses can be extracted appreciably, and noise is greatly suppressed. If the machinery rotates at a constant speed and runs under a single fault condition, usually taking the FFT for this preprocessing signal can obtain the characteristic fault frequency. If the machinery rotates at variable speed or runs under a multisource vibration condition, the impulses will perform their characteristics with aperiodicity and inconsistency. At this time, sparse decomposition can be introduced to extract all the impulses, by which further analysis and diagnosis can be realized. In order to improve the efficiency of the sparse decomposition, a method based on a multi-resolution GST time–frequency spectrum is adopted in this paper.

**Figure 3:**
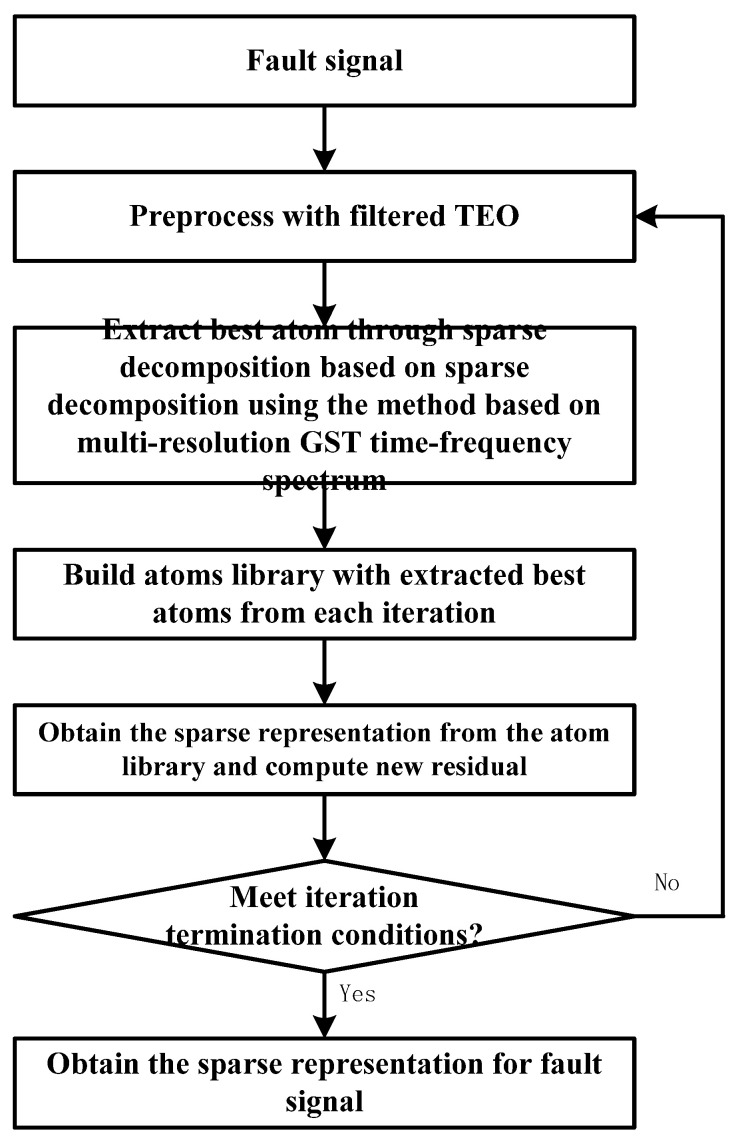
The flow of the proposed sparse decomposition based on the TEO.

The flow of the proposed sparse decomposition based on the TEO is shown in Figure 3.

Realization of this method includes four steps: preprocessing with the filtered TEO to enhance the impulses, extraction of the best atoms for preprocessed signals, reconstruction of atoms for the original fault signal, and sparse representation with reconstructed atoms.

Suppose the time–frequency factors of the extracted atoms from the preprocessed signal are given by

(18) $$\Gamma_{\Psi }=\left\{(\lambda_{\Psi k},f_{k},u_{k},\varphi_{k})|k=1,2,\ldots ,K\right\}$$

where $\Gamma_{\Psi }$ is the set of time–frequency factors for the preprocessed signal $s_{\Psi }(t)$ and K is the number of extracted atoms.

The preprocessed signal can be represented sparsely in the library built from the extracted atoms as

(19) $$s_{\Psi }(t)=\sum_{k=1}^{K}<s_{\Psi }(t),g_{\Gamma_{\Psi k}}>g_{\Gamma_{\Psi k}}$$

where $g_{\Gamma_{\Psi k}}$ is the mathematical model of an atom given by

(20) $$g=\pi^{-\frac{1}{4}}\lambda_{}^{\frac{1}{2}}f_{}^{\frac{1}{2}}e^{-\frac{\lambda_{}^{2}f_{}^{2}{\left(t-u\right)}^{2}}{2}}\cos(2\pi ft+\varphi )$$

Thus, the time–frequency factors of atoms representing the initial fault signal can be calculated by

(21) $$\Gamma =\left\{(\lambda_{k},f_{k},u_{k},\varphi_{k})|\lambda_{k}=\frac{\lambda_{\Psi k}}{\sqrt{3}},k=1,2,\ldots ,K\right\}$$

where Γ is the set of time–frequency factors for the initial fault signal s(t).

The initial fault signal can be represented by

(22) $$s(t)=\sum_{k=1}^{K}<s(t),g_{\Gamma_{k}}>g_{\Gamma_{k}}$$

## 4. Simulation Results and Analysis

The simulation was designed mainly to verify advantages in anti-interference and accuracy. Firstly, we used simulated signals to prove the advantage of using the filtered TEO compared to the traditional TEO. Secondly, we used simulated signals with different SNRs to prove the validity of the proposed method.

### 4.1. Comparison of the TEO and Filtered TEO

Simulated signals were constructed with impulses and white Gaussian noise, which is given by:

(23) $$S=a_{1}S_{p}+a_{2}S_{n}$$

where $S_{p}$ is an impulse with an oscillation frequency of 1000Hz, $S_{n}$ is white Gaussian noise, and $a_{1}$ and $a_{2}$ are the corresponding coefficients.

The simulated signal $S_{1}$ is a noise-free impulse, $S_{2}$ is a noisy impulse, and SNR=−0.71dB. Methods using the TEO, the first derivative of the TEO, the filtered TEO, and the filtered first derivative of the TEO were adopted to process the simulated signals.

**Figure 4:**
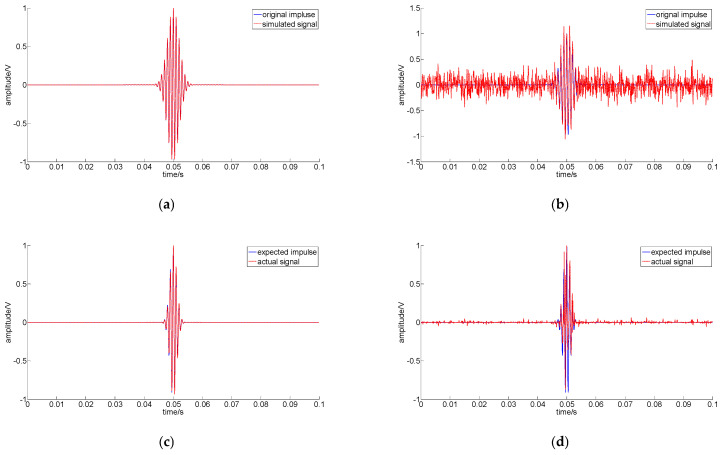

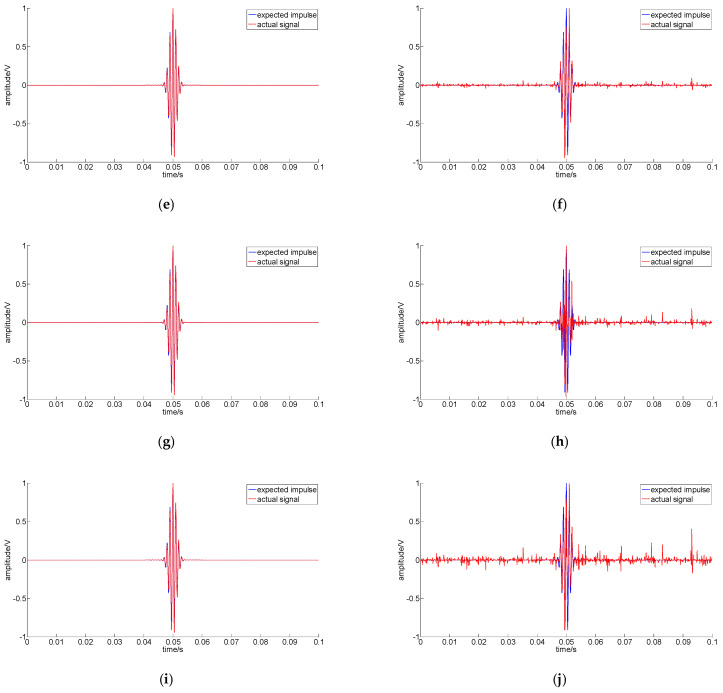
Preprocessed results of *S*_1_ and *S*_2_ with four different TEO methods: (**a**) simulated noise-free signal S_1_; (**b**) simulated noisy signal S_2_, and SNR= −0.71 dB; (**c**) processed using the TEO for S_1_; (**d**) processed using the TEO for S_2_; (**e**) processed using the filtered TEO for S_1_; (**f**) processed using the filtered TEO for S_2_; (**g**) processed using the first derivative of the TEO for S_1_; (**h**) processed using the first derivative of the TEO for S_2_; (**i**) processed using the filtered first derivative of the TEO for S_1_; (**j**) processed using the filtered first derivative of the TEO for S_2_.

Figure 4 shows the preprocessed results of $S_{1}$ and $S_{2}$ with four different TEO methods.

Figure 4a,c,e,g,i shows that all the preprocessed impulses match the expected impulse, which can be computed according to the original impulse. Figure 4b,d,f,h,j shows that all the preprocessed signals enhance the impulse and suppress noise. The comparison of Figure 4d,h shows that the enhanced impulse was affected by high-frequency noise more seriously through the first derivative of the TEO. Figure 4f,j reflects that the filtered TEO and filtered first derivative of the TEO performed better.

To further evaluate the performance of these four methods, correlation of preprocessed impulse and expected impulse was introduced. Obviously, the larger the correlation is, the more similar the preprocessed impulse and expected impulse are, and the better the method performs.

Table 1 shows the correlation of preprocessed impulses and expected impulses with different SNRs.

Table 1 shows that: 1. With a decrease in SNR, the impulse would be covered by noise (column 2); 2. TEO can weakly enhance the impulse in high SNRs, but it is ineffective in low SNRs (column 3); 3. A filtered TEO can greatly enhance the impulse even in very low SNRs (column 4); 4. The first derivative of the TEO cannot enhance an impulse limited by noise (column 5); 5. The filtered first derivative of the TEO can enhance the impulse in high SNRs, but it is also ineffective for low SNRs (column 6).

Therefore, the filtered TEO has the best performance, exceptionally enhancing impulses in very low SNRs. Preprocessing with the filtered TEO can not only retain the structural characteristics of impulses but can also greatly enhance the impulses.

### 4.2. Comparison of the Proposed Method with Traditional Sparse Decomposition Based on Multi-Resolution GST Time–Frequency Spectrum

Three simulated signals consisting of impulses and noises were constructed, which is given by:

(24) $$S=S_{p}+aS_{n}$$

(25) $$S_{p}=\sum_{i=1}^{3}e^{-\frac{\lambda_{i}^{2}f_{i}^{2}{\left(t-u_{i}\right)}^{2}}{2}}\cos(2\pi f(t-u_{i})+\varphi_{i})$$

where $S_{p}$ denotes impulses, $S_{n}$ is white Gaussian noise, and a is the coefficient of noise. To simulate the impulses with aperiodicity and multi-source characteristics, three impulses with different structures were generated, and the time–frequency factors of $S_{p}$ are shown in Table 2.

Changing the value of *a*, simulated signals with different SNRs were generated.$S_{1}$ is a noise-free signal, $S_{2}$ is a noisy signal with SNR = −3.16 dB, and $S_{3}$ is a noisy signal with a lower SNR = −8.75 dB.

**Figure 5:**
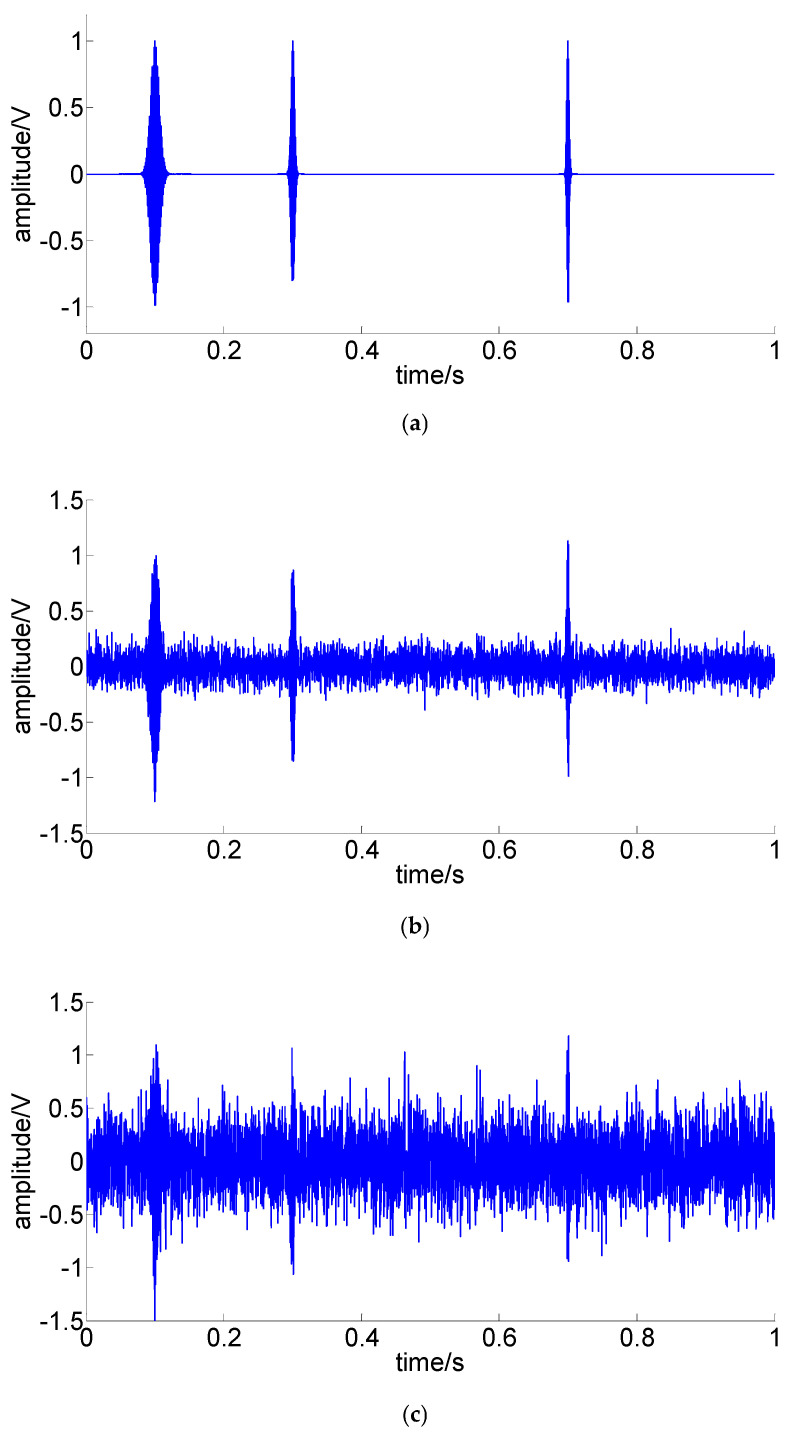
The simulated signals: (**a**) $S_{1}$: noise-free signal; (**b**) $S_{2}$: noisy signal, SNR = −3.16 dB; (**c**) $S_{3}$: noisy signal, SNR = −8.75 dB.

The simulated signals $S_{1}$, $S_{2}$, and $S_{3}$ are shown in Figure 5.

**Figure 6:**
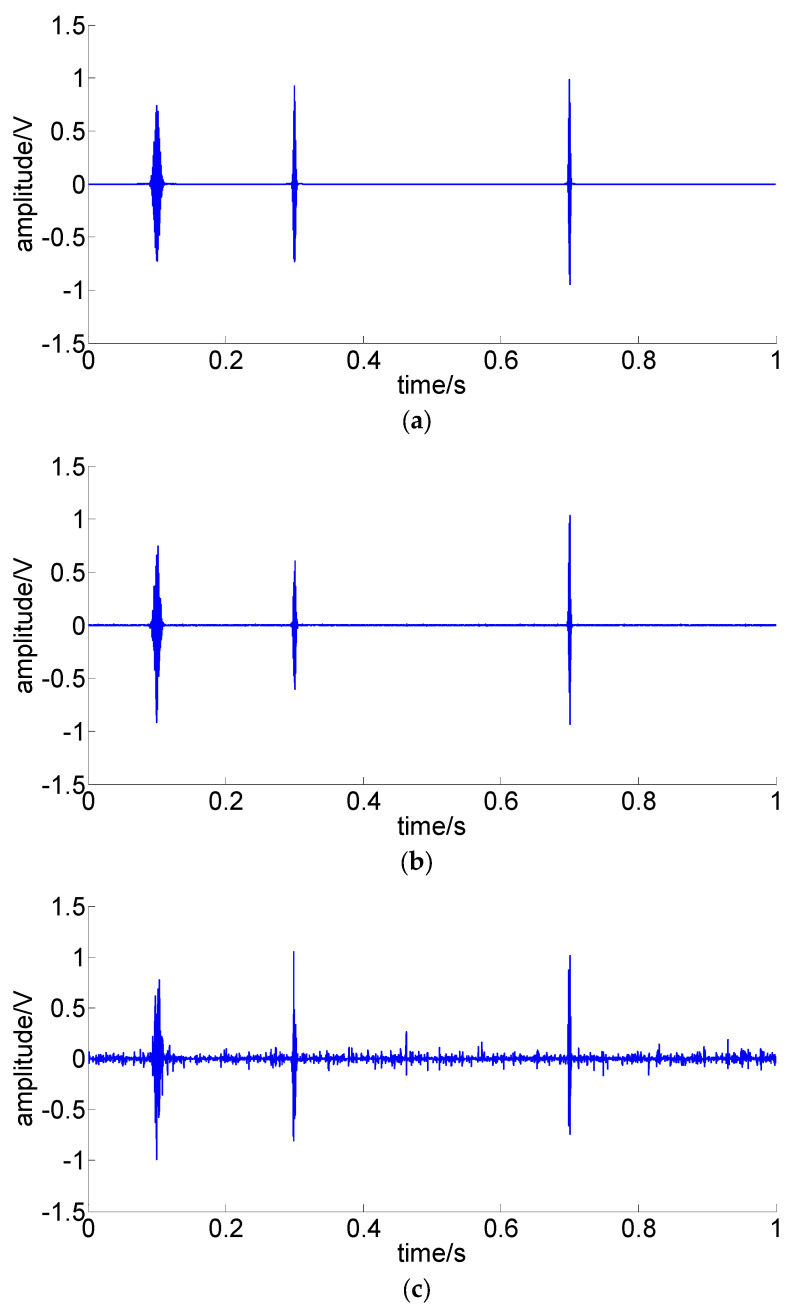
Enhanced signals using the filtered TEO: (**a**) $S_{1}$: noise-free signal; (**b**) $S_{2}$: noisy signal, SNR = −3.16 dB; (**c**) $S_{3}$: noisy signal, SNR = −8.75 dB.

The enhanced versions of the simulated signals using the filtered TEO are shown in Figure 6.

Comparison between Figure 5a and Figure 6a shows the amplified amplitude is related to the impulse frequency and the attenuation of the enhanced signals is more rapid, which matches the characteristics of the TEO. Figure 6b,c shows the impulses are greatly improved.

**Figure 7:**
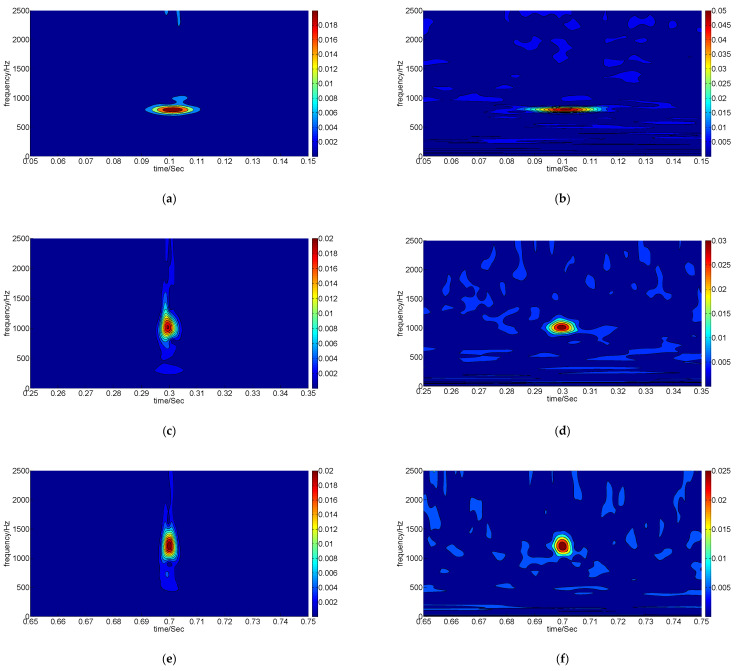
Comparison of proposed method and traditional method: (**a**) first impulse, proposed method, $\lambda =\sqrt{3}*0.197,\;u=0.1002,\;f=800,\;\varphi =0.07\pi$; (**b**) first impulse, traditional method,λ=0.213, u=0.1002, f=801, φ=−0.08π; (**c**) second impulse, proposed method, $\lambda =\sqrt{3}*0.404,\;u=0.2998,\;f=1002,\;\varphi =-0.11\pi$; (**d**) second impulse, traditional method, λ=0.388, u=0.2996, f=1007, φ=0.17π; (**e**) third impulse, proposed method, $\lambda =\sqrt{3}*0.506,\;u=0.7000,\;f=1207,\;\varphi =-0.08\pi$; (**f**) third impulse, traditional method, λ=0.476, u=0.7000, f=1213 φ=−0.12π. The decomposed results are shown in Figure 8.

To describe the good performance of the proposed method, a comparison of the proposed method and traditional sparse decomposition based on a multi-resolution GST time–frequency spectrum for every impulse of *S*_3_ is shown in Figure 7.

**Figure 8:**
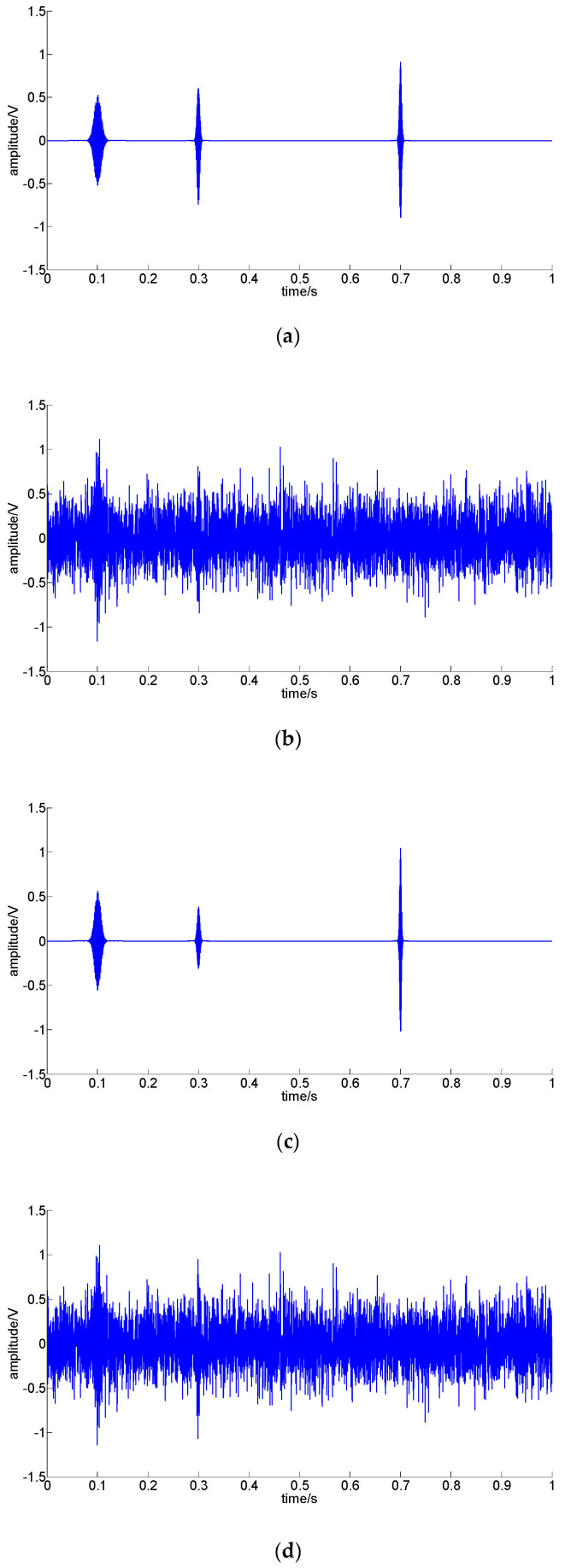
The decomposed results: (**a**) sparse representation, proposed method; (**b**) residual, proposed method; (**c**) sparse representation, traditional method; (**d**) residual, traditional method.

Figure 7a,c,e shows that the optimal spectra with the proposed method can greatly suppress noise by preprocessing through the filtered TEO, and the corresponding λ are close to $\sqrt{3}$ times those of the simulated impulses. Figure 7b,d,f shows that the optimal spectra with the traditional method have strong noise, and the error of time–frequency factors are greater compared to Figure 7a,c,e.

To express the effect of these two methods, Table 3, Table 4, Table 5 and Table 6 show the extracted time–frequency factors for $S_{1}$ and $S_{2}$, respectively.

Table 3, Table 4, Table 5 and Table 6 show that with an increase in noise, the error will increase, which would affect the performance of the sparse representation.

## 5. Engineering Application

**Figure 9:**
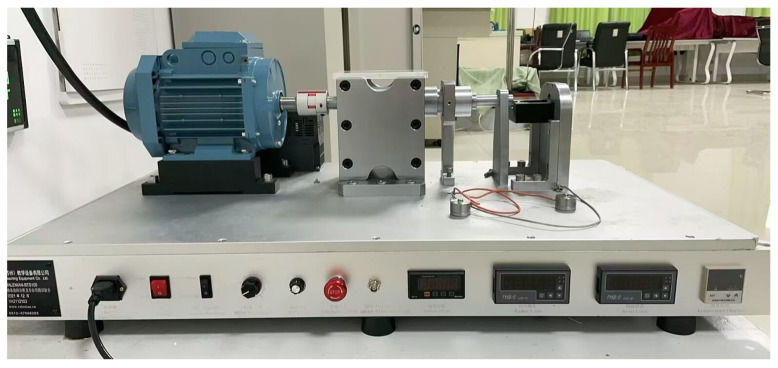
The fault simulation platform.

**Figure 10:**
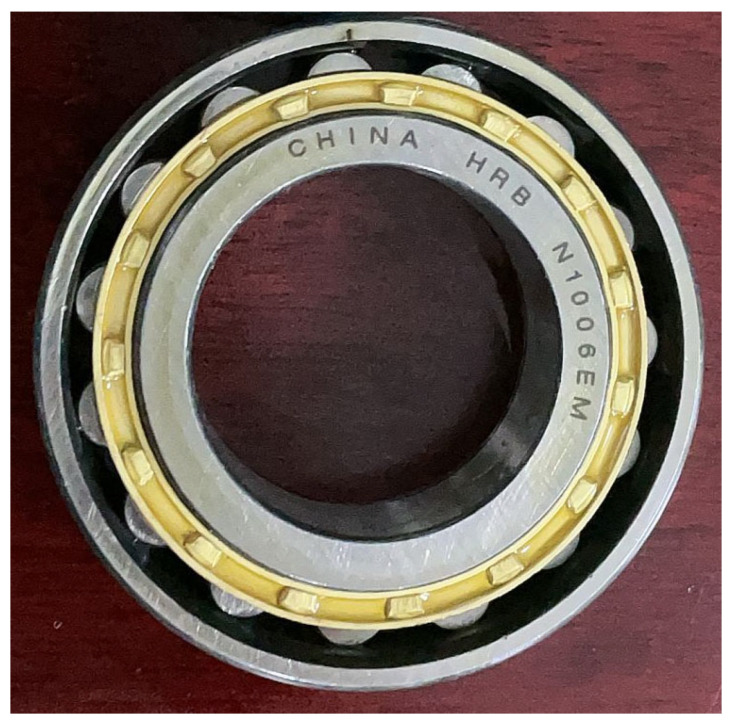
The outer-ring fault bearing.

**Figure 11:**
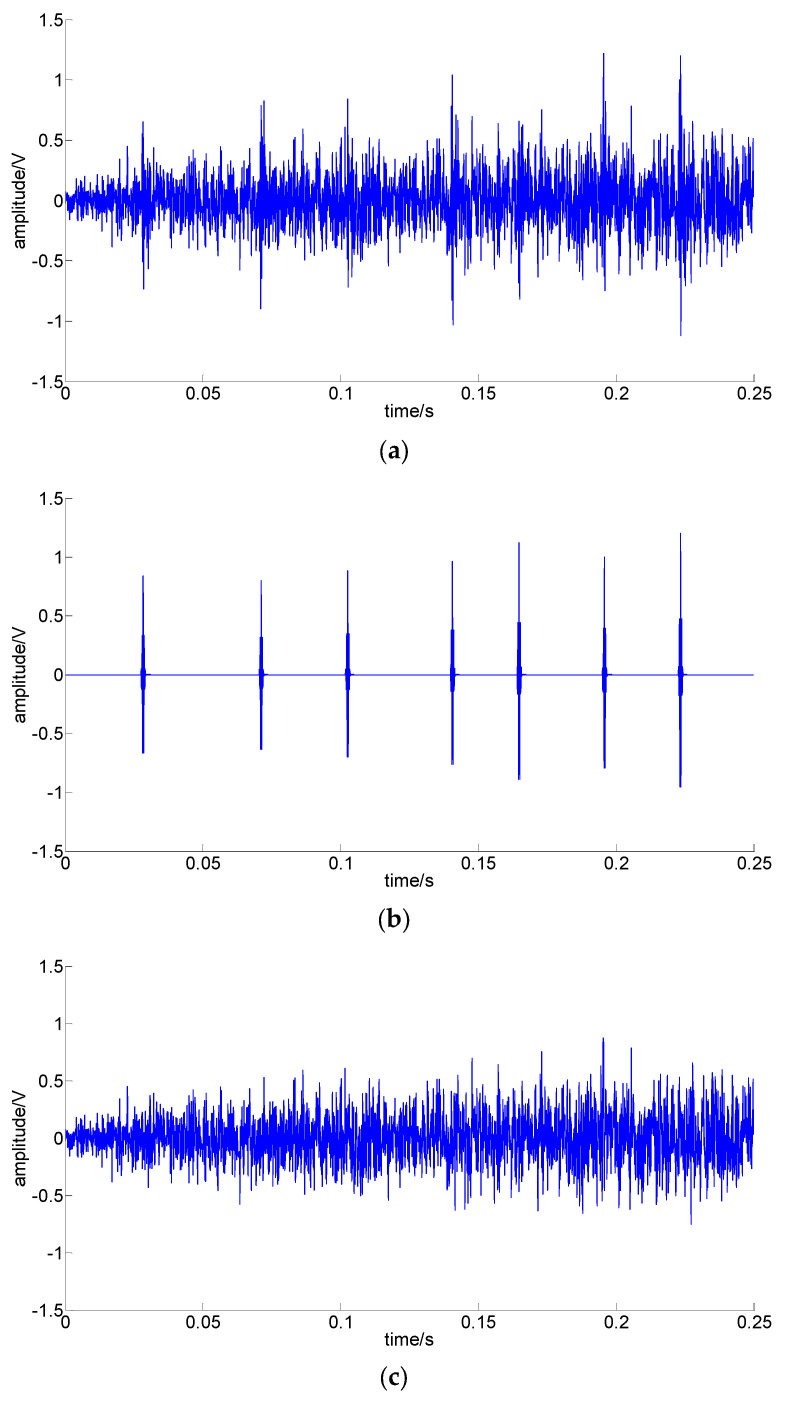
Acquired signal and extracted signal using the proposed method: (**a**) acquired signal; (**b**) extracted impulses; (**c**) residual.

To verify the practicability of the proposed fast sparse decomposition method based on time–frequency spectrum optimization, a set of outer-ring fault signals with rotation speed linear accelerations from 300 r/min to 450 r/min in 0.25 s were acquired from a fault simulation platform for rotating machinery. The fault simulation platform is shown in Figure 9, and the outer-ring fault bearings are shown in Figure 10. The bearing parameters are shown in Table 7. The acquired signal and extracted signal using the proposed method are shown in Figure 11.

Figure 11 shows all the impulses can be extracted from the fault signal, the amplitude is increasing with the rotating speed, and the interval is decreasing with the rotating speed. As the impulses are non-periodic, the traditional frequency analysis method is not applicable. Consistent with the feature of amplitudes and intervals changing with rotation speed, the fault type can be identified as an outer ring fault, which conforms to the facts.

The engineering application proves the proposed method can extract non-periodic impulses exactly and can identify the fault type under conditions of changing rotation speed.

## 6. Conclusions

In this paper, we proposed a fast sparse decomposition based on the Teager energy operator in the extraction of weak fault signals. Results from simulation and engineering applications verify the superiority of the proposed method. Our conclusions are summarized as follows:

(1) The proposed preprocessing method with filtered Teager energy operation is more effective in restraining the low-frequency harmonic component and noise and improving impulses with high oscillation frequency. More importantly, it can retain the structure of the impulse, which is critical to the sparse decomposition.
(2) The proposed sparse decomposition method based on the Teager energy operation performs well in terms of accuracy and efficiency in extracting impulses from low SNR signals and is more applicable in complex and harsh environments.

In the future, we will carry out the following research:

(1) Improve the performance of the filtered TEO, such as an adaptive filtering strategy.
(2) Improve research efficiency in the time–frequency spectra; the proposed research method still needs to generate all the spectra, which need a great deal of calculation.

## Figures and Tables

**Table 1:** Correlation of preprocessed impulses and expected impulses with different SNRs.

SNR (dB)	Simulated Signal	TEO	Filtered TEO	First Derivative of the TEO	Filtered First Derivative of the TEO
22.53	0.9972	0.9976	0.9988	0.9871	0.9968
13.18	0.9766	0.9906	0.9964	0.9260	0.9896
8.54	0.9384	0.9413	0.9825	0.7545	0.9555
2.48	0.8031	0.8245	0.9375	0.4943	0.8691
−0.71	0.6737	0.8172	0.9161	0.4691	0.7883
−3.18	0.5663	0.5150	0.7253	0.2518	0.5246
−5.49	0.4709	0.4091	0.6719	0.2139	0.4977
−7.44	0.3570	0.3016	0.5239	0.1151	0.2893
−9.62	0.2103	0.1992	0.3576	0.1684	0.2281

**Table 2:** Time–frequency factors of $S_{p}$.

No.	Scale Factor λ	Frequency Factor f	Shift Factor u	Phase Factor φ
1	0.2	800	0.1	0
2	0.4	1000	0.3	0
3	0.5	1200	0.7	0

**Table 3:** Time–frequency factors of $S_{1}$ with the proposed method.

No.	Scale Factor λ	Frequency Factor f	Shift Factor u	Phase Factor φ
1	0.200	800	0.1000	0
2	0.400	1000	0.3000	0
3	0.500	1200	0.7000	0

**Table 4:** Time–frequency factors of $S_{1}$ with the traditional method.

No.	Scale Factor λ	Frequency Factor f	Shift Factor u	Phase Factor φ
1	0.200	800	0.1000	0
2	0.400	1000	0.3000	0
3	0.500	1200	0.7000	0

**Table 5:** Time–frequency factors of $S_{2}$ with the proposed method.

No.	Scale Factor λ	Frequency Factor f	Shift Factor u	Phase Factor φ
1	0.203	800	0.1001	0.01π
2	0.395	1001	0.2998	0.02π
3	0.501	1203	0.7001	0.05π

**Table 6:** Time–frequency factors of $S_{2}$ with the traditional method.

No.	Scale Factor λ	Frequency Factor f	Shift Factor u	Phase Factor φ
1	0.208	800	0.1000	0.03π
2	0.381	1003	0.3001	−0.03π
3	0.485	1209	0.7000	−0.07π

**Table 7:** Bearing parameters.

Pitch Diameter D	Roller Diameter d	Roller Number N	Angle α
39.5 mm	7.5 mm	12	0

## References

[1] Lin J.S., Dou C.H. (2015). The diagnostic line: A novel criterion for condition monitoring of rotating machinery. ISA Trans..

[2] Yang Z., Li Z.Q., Zhou F.X., Ma Y.J., Yan B.K. (2022). Weak fault feature extraction method based on improved stochastic resonance. Sensors.

[3] Xu Y.P., Di L., Zhou J., Jin C.W., Guo Q.T. (2016). Active magnetic bearings used as exciters for rolling element bearing outer race defect diagnosis. ISA Trans..

[4] Ma J.P., Jiang J. (2015). Analysis and design of modified window shapes for S-transform to improve time-frequency localization. Mech. Syst. Signal Process..

[5] Osman S., Wang W. (2016). A normalized Hilbert-Huang transform technique for bearing fault detection. J. Vib. Control.

[6] Donoho D.L., Tsaig Y., Drori I. (2012). Sparse solution of underdetermined systems of linear equations by stagewise orthogonal matching pursuit. IEEE Trans. Inf. Theory.

[7] Yan B.K., Zhou F.X. (2015). Initial fault identification of bearing based on coherent cumulant stagewise orthogonal matching pursuit. J. Mech. Eng..

[8] Zhang C., Li Z.X., Hu C., Chen S., Wang J.G., Zhang X.G. (2017). An optimized ensemble local mean decomposition method for fault detection of mechanical components. Meas. Sci. Technol..

[9] Wang L.W., Liu Z.W., Miao Q., Zhang X. (2018). Time-frequency analysis based on ensemble local mean decomposition and fast kurtogram for rotating machinery fault diagnosis. Mech. Syst. Signal Process..

[10] Wu Y.K., Jiang B., Wang Y.L. (2020). Incipient winding fault detection and diagnosis for squirrel-cage induction motors equipped on CRH trains. ISA Trans..

[11] Mallat S.G., Zhang Z.F. (1993). Matching pursuit with time-frequency dictionaries. IEEE Trans. Signal Process..

[12] Yan B.K., Wang B., Zhou F.X., Li W.G., Xu B. (2018). Sparse decomposition method based on time-frequency spectrum segmentation for fault signals in rotating machinery. ISA Trans..

[13] Zhang X., Liu Z.W., Miao Q., Wang L. (2018). Bearing fault diagnosis using a whale optimization algorithm-optimized orthogonal matching pursuit with a combined time-frequency atom dictionary. Mech. Syst. Signal Process..

[14] Teager H.M., Teager S.M. (1983). A phenomenological model for vowel production in the vocal tract. Speech Sci. Recent Adv..

[15] Xu B., Zhou F.X., Li H.P., Yan B.K., Liu Y. (2019). Early fault feature extraction of bearings based on Teager energy operator and optimal VMD. ISA Trans..

[16] Kaiser J.F. On a simple algorithm to calculate the energy of a signal. Proceedings of the IEEE International Conference on Acoustics, Speech, and Signal Processing.

[17] Boudraa A.O., Salzenstein F. (2018). Teager-Kaiser energy methods for signal and image analysis: A review. Digit. Signal Process..

[18] Pei X.L., Zheng X.Y., Wu J.L. (2021). Intelligent bearing fault diagnosis based on Teager energy operator demodulation and multiscale compressed sensing deep autoencoder. Measurement.

[19] Galezia A., Gryllias K. (2021). Application of the Combined Teager-Kaiser Envelope for bearing fault diagnosis. Measurement.

[20] Yan B.K., Wang B., Zhou F.X., Li W.G., Xu B. (2019). Sparse feature extraction for fault diagnosis of rotating machinery based on sparse decomposition combined multiresolution generalized S transform. J. Low Freq. Noise Vib. Act. Control.

